# Supplementary material for: A UMLS-based spell checker for natural language processing in vaccine safety
Source: BMC Med Inform Decis Mak. 2007 Feb 12;7:3. doi: 10.1186/1472-6947-7-3 (PMC1805499; doi:10.1186/1472-6947-7-3)
Supplement: Additional file 13 — RAP application directory. Sets up directory for RAP (RDF application for PHP) source code [file 1472-6947-7-3-S13.gz › rap/doc/tests.html]

RAP - RDF API for PHP Documentation


RDF API Home |
Documentation |
PHPDoc

# Documentation

## Tutorials

1. Introducion to
   RAP
2. Using the statement-centric
   Model API
3. Using the resource-centric
   ResModel API
4. Using the ontology-centric
   OntModel API
5. Using the RDF Dataset API
6. RDQL tutorial
7. Database
   Persistence
8. RDF DB Utils tutorial
9. GRDDL Parser
10. Using vocabularies
11. Graph visualization

## API Documentation

1. PHPDoc

## Implementation Notes

1. Database Backend
2. RDQL Engine

## Usage Examples

1. Manipulate and Seach MemModel

- HTML output
  (including source code)
- PHP source
  code

2. Traverse MemModel

- HTML output (including
  source code)
- PHP source code

3. Using the InfModel

- HTML output (including
  source code)
- PHP source code

4. Using the ResModel
   - HTML output (including
     source code)
   - PHP source code
5. Using the DatasetMem API
   - HTML output (including
     source code)
   - PHP source code
6. Set Operations

- HTML output (including
  source code)
- PHP source code

7. Serializer Options

- HTML output
  (including source code)
- PHP source
  code

8. Custom API Test

- Test the API with your own RDF code: Interactive
  online demonstration.

9. Set Database Connection

- HTML output
  (including source code)
- PHP source
  code

10. Storing Models in a Database

- HTML
  output (including source code)
- PHP source
  code

11. Manipulate and Seach Database Model

- HTML output
  (including source code)
- PHP source
  code

12. Query a Model using the RDQL Query Language

- HTML output (including
  source code)
- PHP source code

13. Custom RDQL Test

- Test the RDQL query engine using your own RDF model
  and your own queries: Interactive
  Query demonstration
